# Modern Medicine Is Neglecting Road Traffic Crashes

**DOI:** 10.1371/journal.pmed.1001463

**Published:** 2013-06-11

**Authors:** Donald A. Redelmeier, Barry A. McLellan

**Affiliations:** 1 Sunnybrook Health Sciences Centre, University of Toronto, Toronto, Ontario; 2 Department of Medicine, University of Toronto, Toronto, Ontario; 3 Evaluative Clinical Sciences, Sunnybrook Research Institute, Toronto, Ontario; 4 Institute of Health Policy Management & Evaluation, University of Toronto, Toronto, Ontario; 5 Institute for Clinical Evaluative Sciences in Ontario, Toronto, Ontario; 6 Department of Surgery, University of Toronto, Toronto, Ontario

## Abstract

Don Redelmeier and Barry McLellan admonish the medical community for failure to act on the vast problem of road traffic crashes.

*Please see later in the article for the Editors' Summary*

**Summary Points**

- Despite a high burden of disease, motor vehicle trauma has not generated proportionate attention in clinical medicine, public health agencies, or the wider community.
- The relative neglect arises in part from a denial of aggregate statistics, the banality of everyday crashes, and the lack of a labelled lobby group.
- Further clinical factors include biased beliefs among surviving patients, segregation of clinical services, inescapable research limitations, and idiosyncratic scientific traditions.
- Additional policy barriers arise from conflicting economic priorities, fundamental insuperability, and inescapable cultural diversity.
- Significant progress toward mitigating motor vehicle trauma will likely continue by modest positive incremental gains in road safety.

## Introduction

Traffic crashes are a common cause of human suffering, projected to become the fifth leading cause of death in the year 2030 [1]. The current annual worldwide losses amount to about 1.2 million fatalities, 20 million patients surviving with disabilities, and 100 million persons with economic losses from property destruction [2]. Surprisingly, the high burden of traffic crashes remains neglected in medical textbooks, MEDLINE citations, and health research funding agencies [1],[3]. The paradoxical mismatch between relative importance and relative inattention has led to repeated calls for changes to promote more public health protection [4]–[7]. The purpose of this Essay is to highlight (and juxtapose with counterexamples) factors that underpin the relative neglect of traffic crashes as a cause of patient mortality and morbidity.

## Denial of Aggregate Statistics

Most vehicle trips do not result in a crash, thereby leaving the intuitive impression that such activity is safe and that the statistics are lying. Even trips with multiple infractions (e.g., speed violations, improper turns, alcohol intoxication) rarely result in an adverse outcome. As such, years of experience as a motorist tend to reinforce a false sense of security and a mistaken belief that traffic risks are not personally salient. A widespread disregard for risks is further encouraged by popular movies, television shows, computer games, and industry advertising wherein perilous driving rarely leads to adverse outcomes for a heroic protagonist [8]. In contrast, most people interpret statistics related to heart disease with genuine interest and believe that these risks will eventually be personally important.

## Banality of Everyday Life

Traffic crashes are so frequent in the general community that the problem is also somewhat banal (about 80,000 total crashes daily worldwide). Any immersive exposure inevitably can lead to a culture of complacency because each incident seems a routine rather than an exotic newsworthy event [9]–[12]. Furthermore, the seriousness of many reported traffic crashes is ambiguous because of the proportion of traffic crashes that result in property damage without human injury. A glib nihilistic response is also possible because each reported fatal incident usually has a counterpoint of a different motorist who had no mishap on the same road on the same day. In contrast, deaths due to Ebola virus are compelling because each case is rare, no arguments arise about severity, and the counterfactual is not instantly imagined.

## Lack of a Labelled Lobby Group

The high speed of traffic implies that the time interval between incident and injury is less than 1 second. Many patients, therefore, become immediately debilitated and unable to advocate for their own well-being or activate a philanthropic donor [13]. In turn, politicians and regulators rarely encounter public campaigns, professional lobbying, or mainstream journalism from those injured in crashes. One major exception for advocacy in the United States is the group Mothers Against Drunk Driving (MADD), which succeeds by addressing one factor (impaired driving) and one population (young road users). In contrast, activism around prostate cancer has been much more effective at raising public attention because a positive prostate-specific antigen (PSA) test allows patients to identify their situation for many years before becoming disabled.

## Biased Beliefs Among Survivors

Road users who recover from a serious crash often persist with faulty beliefs despite their objective personal evidence of traffic dangers. Among survivors, for example, a natural reaction is to blame others and to attribute the crash to surrounding drivers or circumstances. Even survivors of single vehicle crashes can readily attribute their incident to some idiosyncratic condition that might be entirely avoided in the future [14]. Furthermore, some survivors have a history of antisocial personality disorder, anomalous lifestyle choices, chronic substance abuse, or other potentially unappealing attributes [15]. In contrast, breast cancer survivors are more likely to become grateful outspoken public champions who advocate effectively for future improvements.

## Segregation of Clinical Services

Motor vehicle trauma patients tend to receive care in a few elite hospitals designated as trauma centers. Other clinicians in the community, therefore, become unfamiliar with such cases and easily underestimate the overall burden of disease. Clinicians practicing in trauma centers will be fairly aware of the overall burden, but might be demoralized by attendant clinical frustrations (e.g., brain injury is the most common serious complication and yet has no reliable cure). The amount of clinical enthusiasm is further attenuated because the most effective interventions for motor vehicle trauma emphasize primary prevention and smack of public health restrictions that curb personal freedom. In contrast, patients with mental health disorders garner more attention in the wider medical community, are prevalent across diverse clinical settings, seek care because of symptoms, and can be treated with multiple effective interventions [16].

## Inescapable Research Limitations

Motor vehicle travel is a human activity that cannot be mimicked by animal models or molecular biological techniques. As a consequence, the experimental methods that lead to amazing advances in basic medical science contribute only indirect insights for traffic crashes, if they apply at all. Research around motor vehicle trauma also lacks many of the strengths of clinical science, such as randomized interventions, blinded participants, independent observations, long-term follow-up, and standardised outcomes. The main exceptions are simulator studies, yet this approach raises further criticisms related to volunteer participants engaging in artificial tasks with hypothetical risks. In contrast, patients with Crohn's disease, for example, have benefitted from the accumulation of biological insights grounded in basic science that lead to powerful monoclonal antibody treatments.

## Idiosyncratic Scientific Traditions

High-quality research science is also stymied because of the fallibilities of human scientists. Individual trauma case reports are dramatic, emotional, and readily understood in a manner that may sometimes squelch the wonderment needed for scientific inquiry. Professional conferences for trauma surgeons or traffic engineers commonly provide startling photographs of wrecked vehicles or disrupted human anatomy that accurately describe the event yet indirectly distract an audience from seeing a larger abstract picture. Another limitation may be that trauma surgeons are sometimes busy late at night and have less time available for prolonged scientific deliberation. In contrast, researchers focusing on diabetes mellitus, for example, are rarely engrossed with acute case reports, dramatic graphical images, or flagrant mechanical explanations.

## Conflicting Economic Priorities

Vehicle travel is generally a substantial positive contributor to quality of life and overall economic prosperity. Hence, misconceived interventions to improve traffic safety could easily cause unanticipated consequences, an increase in expenditures, and a net loss of individual well-being (e.g., unfairly removing driving licenses from healthy seniors who require a car for their daily activities). This tension between safety and freedom implies that an integrated medical perspective is not wholly appropriate for guiding road safety interventions. A larger multidisciplinary approach (to promote active transport, public transit, changes in infrastructure, and alternative land use) is more important— yet also more complex — than for other adverse behaviours, such as smoking or obesity. In contrast, interventions for treating dementia have the promise of directly improving patient well-being, increasing economic prosperity, and invoking no ambiguities around medical legitimacy.

## Fundamental Insuperability

The principal cause of traffic injury is energy, which is also fundamental for almost all human activity. The net contribution from road safety, therefore, is a reduction in (but not elimination of) the frequency of misadventures related to human error. People, however, tend to assign less value to a partial risk reduction (even if the baseline rate is high) and a higher priority to a complete risk removal (even if the baseline rate is low) [17]. The disenchantment around marginal gains is further exacerbated because of the relative absence of simple clinical interventions and endpoints analogous to prescribing a statin medication to treat a patient's hypercholesterolemia. In contrast, eradicating a disease like smallpox is theoretically possible and would be heralded as a realistic milestone in human history [18].

## Inescapable Cultural Diversity

All traffic crashes are local events that reflect the specifics of a particular road, motorist, and vehicle. The causes and consequences of each incident, therefore, are context dependent and not fully generalizable across different settings. To be sure, some insights about road safety in high-income countries may also be applicable in low-income countries (and vice versa) [19]. Yet differences in baseline rates, human factors, vehicle characteristics, roadway designs, infrastructure, public health policies (i.e., to promote walking, cycling, and public transit), and surrounding culture imply that a single finding may not apply equally across even small regions of the same country [20]. In contrast, patients with appendicitis are sufficiently similar throughout the world so that science can generate a unifying force leading to universal truths.

## Conclusion

Predictions of the future are always fallible because of the difficulty in anticipating unforeseeable events. Yet these 10 principles, we believe, are enduring concepts around motor vehicle trauma that will prevail into the years ahead. Indeed, all persisted despite the death of Princess Diana in a crash 15 years ago- an unforeseen incident that generated global attention yet did not galvanize changes in motor vehicle safety. It is hard to imagine another future event that will attract more attention toward road trauma as a neglected non-communicable disease. Fantasizing about large increases in awareness may lead to subsequent disillusionment. Instead, we lay out a pragmatic vision (Boxes 1 and 2) to promote slow, positive incremental gains around motor vehicle crashes in a world fighting for peoples' attention.

**Box 1. Policy Recommendations to Improve Motor Vehicle Safety**

- Continue to emphasize motor vehicle trauma as an ongoing public health epidemic through work by the WHO and other agencies.
- Apply insights from behavioural decision science to mitigate motor vehicle trauma; most crashes can be prevented by a small change in driver behaviour.
- Explore and address the relative shortfall in philanthropic support from large manufacturing industries.
- Ensure strategies to prevent motor vehicle trauma are informed by science and implemented with stakeholder involvement of community values.

**Box 2. Clinical Steps to Improve Motor Vehicle Safety**

- Treat seizures, syncope, mania, and other debilitating conditions that would otherwise make the patient at risk for a traffic crash.
- Emphasize cautions against driving when treating patients acutely with narcotics, sedatives, brain radiation, or other interventions that cause short-term impairments.
- Give medical warnings to patients who have uncontrolled alcoholism, sleep apnea, or other chronic diseases that make the patient unfit to drive.
- Consider use of informal assessment tools that are available for screening indeterminate patients for fitness to drive, taking into account the effectiveness of such prevention strategies is uncertain.
- Follow acute resuscitation protocols in the aftermath of a serious traffic crash and extend to include counselling of survivors to prevent trauma recidivism.
